# Author Correction: Inactivation of cytidine triphosphate synthase 1 prevents fatal auto-immunity in mice

**DOI:** 10.1038/s41467-025-67498-7

**Published:** 2025-12-19

**Authors:** Claire Soudais, Romane Schaus, Camille Bachelet, Norbert Minet, Sara Mouasni, Cécile Garcin, Caique Lopes Souza, Pierre David, Clara Cousu, Hélène Asnagli, Andrew Parker, Paul Palmquist-Gomes, Fernando E. Sepulveda, Sébastien Storck, Sigolène M. Meilhac, Alain Fischer, Emmanuel Martin, Sylvain Latour

**Affiliations:** 1 Laboratory of Lymphocyte Activation and Susceptibility to EBV infection, Inserm UMR 1163, Institut Imagine, Paris, France; 2 Université de Paris Cité, Paris, France; 3 Laboratory of Molecular Basis of Altered Immune Homeostasis Inserm UMR 1163, Institut Imagine, Paris, France; 4 Transgenesis Platform, Laboratoire d’Expérimentation Animale et Transgenèse (LEAT), Institut Imagine-Structure Fédérative de Recherche Necker INSERM US24/CNRS, Paris, France; 5 Université Paris Cité, CNRS UMR 8253, INSERM U1151, Institut Necker Enfants Malades, Paris, France; 6 Step-Pharma, Technoparc du Pays-de-Gex, Saint-Genis-Pouilly, France; 7 Imagine - Institut Pasteur, Unit of Heart Morphogenesis, INSERM UMR1163, Paris, France; 8 Collège de France, Paris, France

**Keywords:** Lymphocytes, Immunological disorders

Correction to: *Nature Communications* 10.1038/s41467-024-45805-y, published online 04 March 2025

In the version of the article initially published, due to a figure preparation mistake in Fig. 7d, the left-hand image in the “Liver” row was a duplicate of the right-hand image. The figure is now amended in the HTML and PDF versions of the article.

