# Supplementary material for: Inactivation of cytidine triphosphate synthase 1 prevents fatal auto-immunity in mice
Source: Nat Commun. 2024 Mar 4;15:1982. doi: 10.1038/s41467-024-45805-y (PMC10912214; doi:10.1038/s41467-024-45805-y)
Supplement: Supplementary file 1 — Supplementary Information [file 41467_2024_45805_MOESM1_ESM.pdf]

Supplementary Data

Inactivation of cytidine triphosphate synthase 1 prevents fatal auto-immunity in mice.

Claire Soudais[1,2*], Romane Schaus[1], Camille Bachelet[1,2], Norbert Minet[1,2], Sara Mouasni[3], Cécile Garcin[1,2], Caique Lopes Souza[1,2], Pierre David[4], Clara Cousu[5], Hélène Asnagli[6], Andrew Parker[6], Paul Palmquist-Gomes[2,7], Fernando E. Sepulveda[3], Sébastien Storck[5], Sigolène M. Meilhac[2,7], Alain Fischer[1,8#], Emmanuel Martin[1#] and Sylvain Latour[1,2*]

Corresponding authors: Claire Soudais, claire.soudais@inserm.fr and Sylvain Latour, sylvain.latour@inserm.fr

Supplementary information

To better characterize the role of CTPS1 *in vivo*, we first developed a mouse model mimicking the human deficiency. Human CTPS1 deficiency is caused by a G>C transversion at the acceptor splicing site of exon 18 leading to the skipping of exon 18 (**Supplementary Data Fig. 1a**). The same G>C was introduced by CRISPR-Cas9 in mice genome and homozygous animals for the mutation (*Ctps1*$^{ki/ki}$) were obtained (**Supplementary Fig. 1b**). cDNA from *Ctps1*$^{ki/ki}$, *Ctps1*$^{wt/ki}$, *Ctps1*$^{wt/wt}$ were analysed and sequenced. Data show, in *Ctps1*$^{ki/ki}$, a deletion of 12 nucleotides (**Supplementary Fig. 1d**). When analysed, the homozygote mutated allele was expressed at the same level than the wild-type allele, in contrast to the human mutated allele that resulted in a strongly decreased expression of CTPS1 expression (**Supplementary Fig. 1d, Martin, E. et al., 2014**). Moreover, T cell proliferation of enriched splenic T cells from *Ctps1*$^{ki/ki}$ with anti-CD3/CD28 beads plus IL-2 was normal and comparable to that of T cells from *Ctps1*$^{wt/ki}$ (**Supplementary Fig. 1e**). We concluded that the *Ctps1*$^{ki/ki}$ mouse did not mimic the human mutation and was thus not a model for *Ctps1* deficiency. Our observation shows the use of a cryptic splicing site leading to a truncated cDNA. This is likely because splicing constraints apply differently in mice compared to humans not resulting the skipping of exon18 in mice.

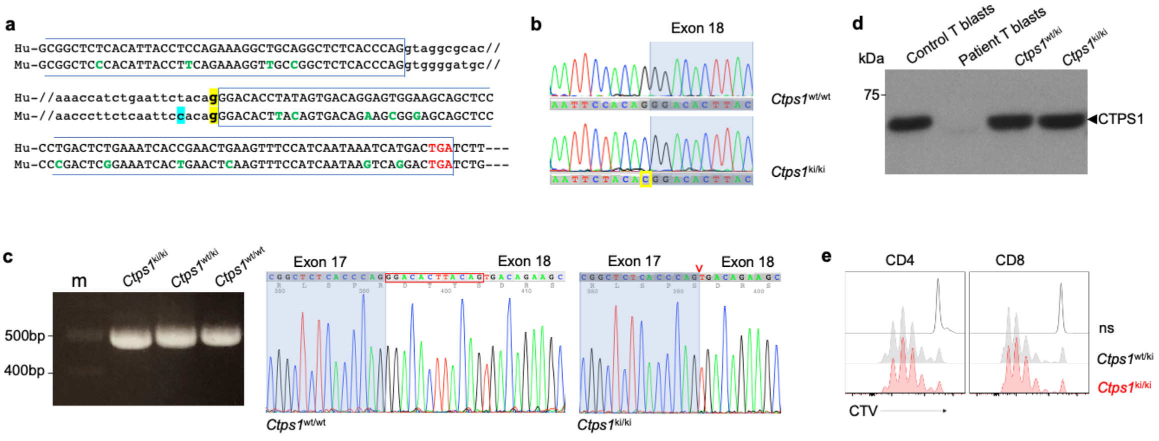

Supplementary Figure 1. **Generation and analysis of knock-in *Ctps1* mice mimicking the human mutation causing CTPS1 immunodeficiency.**

(a) Alignment of human (upper lane) and mouse (lower lane) *CTPS1* sequences focused on exon 17, intron 17 and exon 18 as indicated. Nucleotides differences between human and mouse are in bold green, exons are in upper case letters, introns in lower case letters, the splice site mutation is highlighted in yellow, silent CRISPR-Cas9 mutation is highlighted in blue, stop codon are in red. The mutation G to C causing CTPS1 deficiency in human is highlighted. **(b)** Sequences showing the genotypes of wild type animal (*Ctps1*$^{wt/wt}$) and animals in which the equivalent human mutation was introduced (*Ctps1*$^{ki/ki}$). Mutation is highlighted in yellow. **(c)** PCR analysis of Ctps1 cDNA prepared from *Ctps1*$^{ki/ki}$, *Ctps1*$^{wt/ki}$ and *Ctps1*$^{wt/wt}$ animals (left panel). cDNA sequences from *Ctps1*$^{ki/ki}$ and *Ctps1*$^{wt/wt}$ animals showing the effect of the splice site mutation. **(d)** Western Blot of total spleen lysates from *Ctps1*$^{wt/ki}$ and *Ctps1*$^{ki/ki}$ animals compared with lysates from T blasts of a healthy donor or a patient with CTPS1-deficiency. **(e)** Representative proliferation histogram profiles from FACS analysis of enriched spleen T cells labelled with cell trace violet (CTV) and activated or not with anti-CD3/CD28 beads plus IL-2 for 3 days. Gating on CD4^{+} and CD8^{+} T cells in left and right panels respectively. Grey and red histograms correspond to *Ctps1*$^{wt/ki}$ and *Ctps1*$^{ki/ki}$ respectively. White histograms represent unstimulated cells that did not divide.

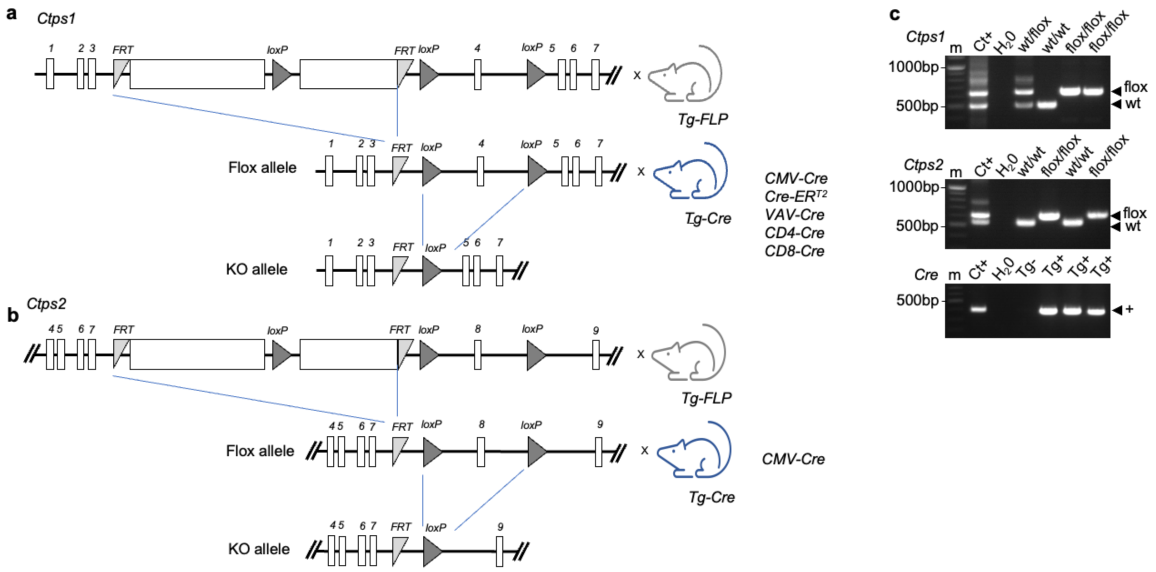

Supplementary Figure 2. **Strategy to obtain conditional mice deficient for *Ctps1 and Ctps2.***

(a) KOMP original constructs and strategy to obtain *Ctps1* Flox alleles by crossing with Flippase-transgenic animals (Tg-*FLP*) that remove the cassette between the two FRT sites. *Ctps1*$^{\text{flox/flox}}$ animals then crossed to specific-promoter transgenic (Tg-*Cre*) animals resulting in Tg-*Cre; Ctps1*$^{\text{flox/flox}}$ (*Ctps1*$^{\text{ko/ko}}$) animals. **(b)** EUCOM original constructs and strategy to obtain *Ctps2* Flox alleles by crossing with Flippase-transgenic animals (Tg-*FLP*) that remove the cassette between the two FRT sites. *Ctps2*$^{\text{flox/flox}}$ animals then crossed to specific-promoter transgenic (Tg-*Cre*) animals resulting in Tg-*Cre; Ctps2*$^{\text{flox/flox}}$ (*Ctps2*$^{\text{ko/ko}}$) animals. **(c)** Example of PCR results from DNA screening of ear biopsies of *Ctps1, Ctps2,* and Tg-*Cr*e.

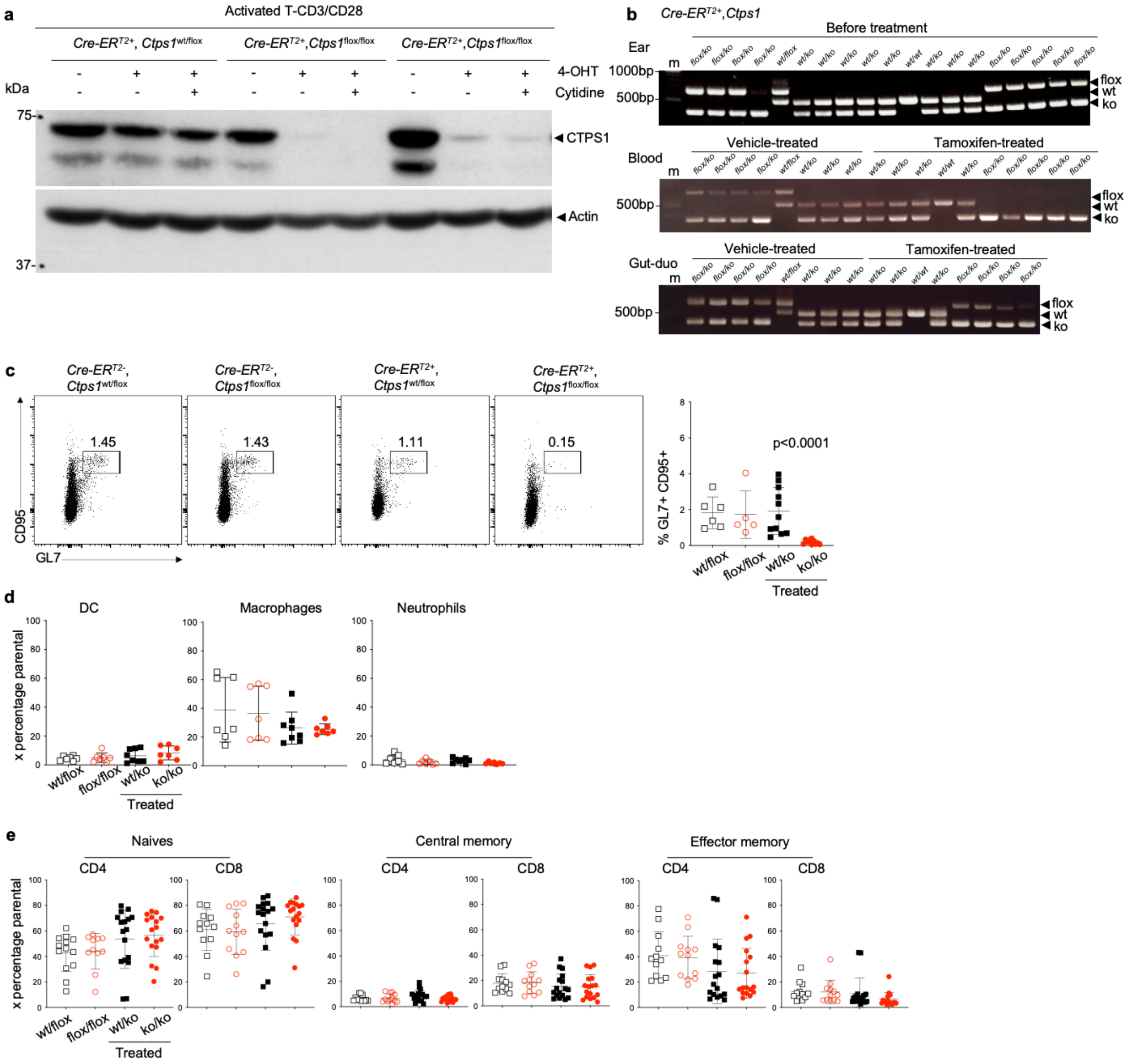

Supplementary Figure 3. Consequences of inducible CTPS1 deficiency.

(a-e) Complementary analysis *Cre-ER*^{T2}; *Ctps1*$^{wt/flox}$ (*Ctps1*$^{wt/ko}$) and *Cre- ER*^{T2}; *Ctps1*$^{flox/flox}$ (*Ctps1*$^{ko/ko}$).

(a) Immunoblots for CTPS1 and Actin expression in lysates of *in vitro* activated T cells non-treated or incubated with 4-OHT plus or minus cytidine. One *Cre- ER*^{T2}; *Ctps1*$^{wt/flox}$ and two *Cre- ER*^{T2}; *Ctps1*$^{flox/flox}$ animals are shown. **(b)** Genotyping by PCR of ear punch before *in vivo* tamoxifen treatment (upper panel), and blood (middle panel) and gut (lower panel) after tamoxifen treatment at day 14 of sacrifice. Corresponding amplified alleles/genotypes are indicated on the left (flox, wt and ko). **(c)** Representative dot-plots from FACS analysis of Peyer patches for non-treated and tamoxifen treated animals, stained with anti-CD95 and anti-GL7. Left panels: percentages of germinal centre (GC) B cells in all genotypes. **(d)** Percentages of splenic dendritic cells, macrophages and neutrophils calculated from FACS analyses. **(e)** Proportions of T cell subsets from FACS analyses. Naive T cells (CD44^{-}CD62L^{+}), central memory (CD44^{+}CD62L^{+}), effector memory (CD44^{+}CD62L^{-}) are shown in both CD4^{+} and CD8^{+} T cells. Non-parametric Matt-Whitney two-tailed test were used. Each individual dot is the value from individual animal. Error bars represent the mean ± SD of n=6 (wt/flox), n=5 (flox/flox), n=11 (wt/ko) and n=10 (ko/ko) animals per group (**panel c**). Data are from n=7 (wt/flox), n=7 (flox/flox), n=8 (wt/ko) and n=7 (ko/ko) animals per group (**panel d**) and n=12 (wt/flox), n=11 (flox/flox), n=17 (wt/ko) and n=17 (ko/ko) animals per group (**panel e).**

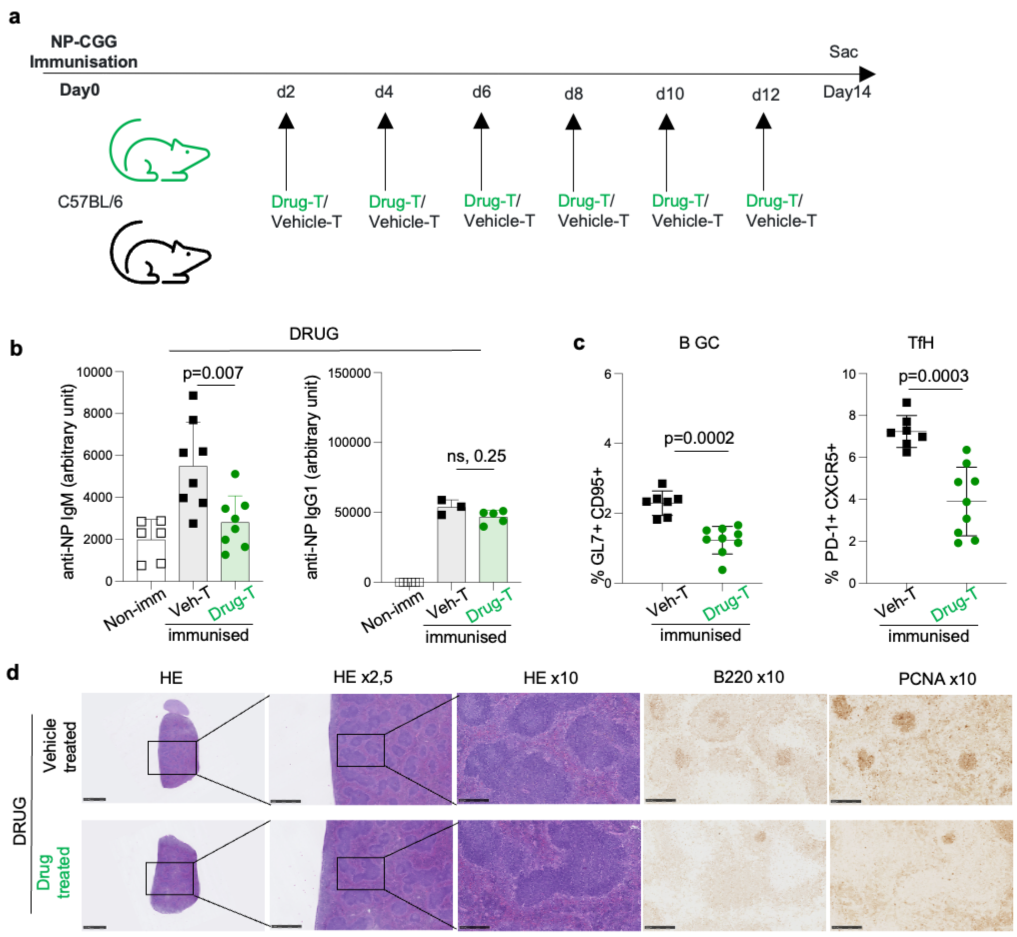

Supplementary Figure 4. Complementary analyses of antibody responses after immunisation in CTPS1 deletion animals or after CTPS1 inhibition.

(**a-d**) Complementary analyses of antibody responses after immunisation of C57BL/6 animals treated with a compound targeting CTPS1.

(a) Experimental design: C57BL/6 animals were immunised with NP-CGG at day 0. C57BL/6 animals were further injected with the inhibitor of *Ctps1* (green) or vehicle (black) every two days. **(b)** NP-specific IgM (left), and IgG1 (right) antibodies, from non-immunized C57BL/6 animals and immunised C57BL/6 animals injected with the vehicle or the inhibitor of *Ctps1*, were quantified by ELISA (arbitrary unit). **(c)** Percentages of germinal centre (GC) B cells, anti-CD95 and anti-GL7 (left) and T follicular helper cells (Tfh), anti-CXCR5 and PD-1 (right) obtained from dot plots of C57BL/6 animals injected with the inhibitor of *Ctps1* or vehicle. (**d**) Representative microscopy images of haematoxylin-eosin coloration, B220 and PCNA labelling of spleen from C57BL/6 animals injected with the inhibitor of *Ctps1* or vehicle. Images are representatives of three animals per group. (Scale bars from left to right: 5mm, 1mm and 250µm). (**panel b, c**) Non-parametric Matt-Whitney two-tailed test were used. Bar chart and error bar represent mean with ± SD of n=6 (non-immunised); n=8 (vehicle treated) and n=8 (drug treated) animals per group (**panel b**) and error bars represent the mean ± SD of n=7 (vehicle treated) and n=9 (drug treated) animals per group (**panel c**).

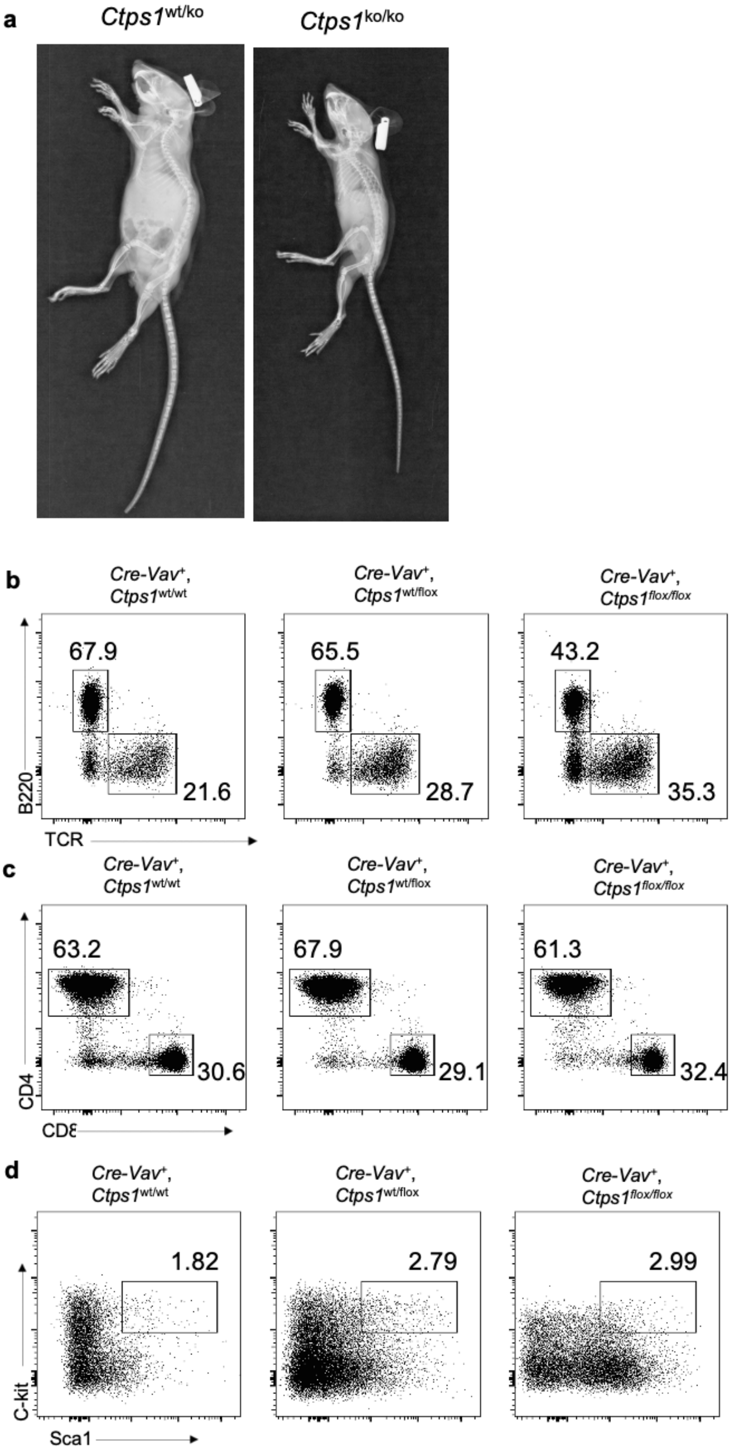

Supplementary Figure 5. Complementary analyses of Ctps 1 inactivation upon the hematopoietic specific promoter VAV.

(a-d) Complementary analyses of of *VAV-Cre*; $Ctps1^{wt/flox}$ ($Ctps1^{wt/ko}$) and *VAV-Cre*; $Ctps1^{flox/flox}$ ($Ctps1^{koko}$) animals. **(a)** Representative radiography done on whole skeletons using a Faxitron (Tucson, AZ, USA) MX-20 DC12, to assess a possible bone defect. **(b-c)** Representative dot-plots of FACS analyses of splenocytes stained with anti-B220 and anti-TCR antibodies **(b)** and T cells stained with anti-CD4 and anti-CD8 antibodies **(c)**. **(d)** Representative dot-plots of FACS analyses of bone marrow cells stained with anti-c-kit and anti-Sca1 to identify LSK- stem cell progenitors.

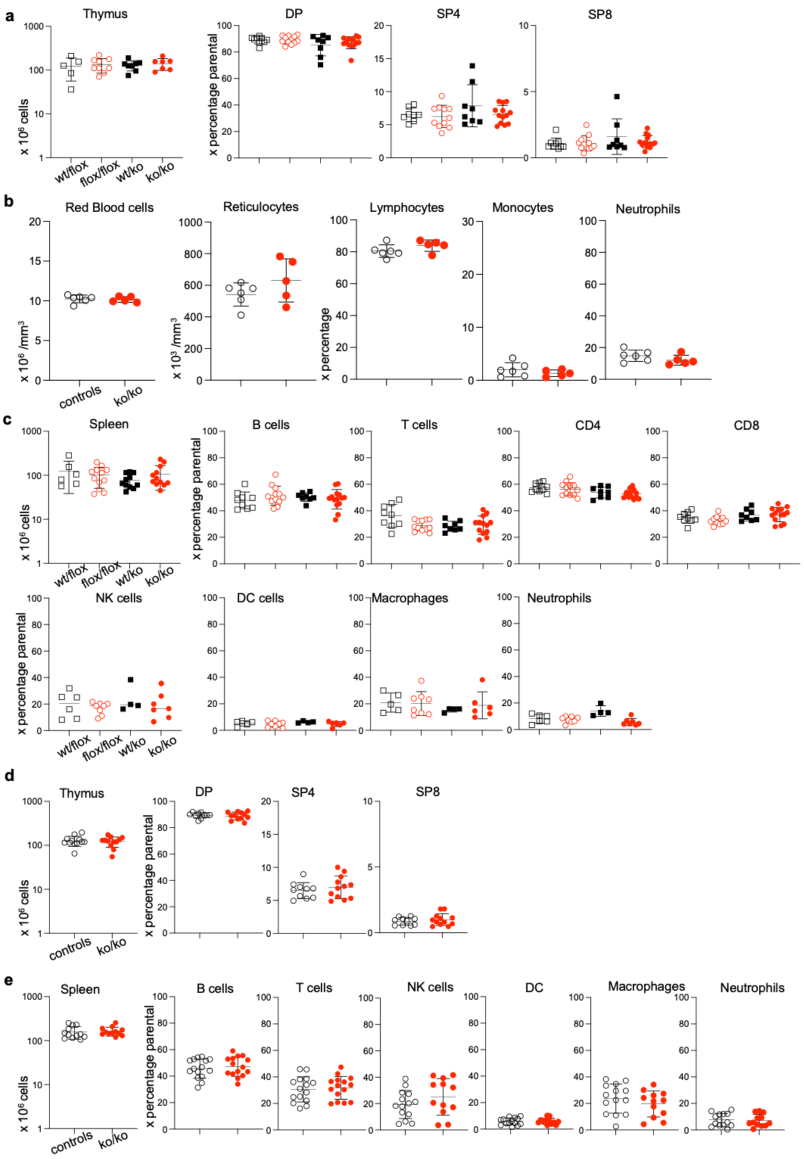

Supplementary Figure 6. Complementary analyses of *Ctps1* inactivation upon the *CD4* or the *CD8* promoter.

Complementary analyses of *CD4-Cre*; *Ctps1*$^{\text{wt/flox}}$ (*Ctps1*$^{\text{wt/ko}}$) and *CD4-Cre*; *Ctps1*$^{\text{flox/flox}}$ (*Ctps1*$^{\text{ko/ko}}$) **(a-c)** and *CD8-Cre*; *Ctps1*$^{\text{wt/flox}}$ (*Ctps1*$^{\text{wt/ko}}$) and *CD8-Cre*; *Ctps1*$^{\text{flox/flox}}$ (*Ctps1*^{koko}) **(d-e)**.

(a) Total thymus cell counts (left panel) and proportions (right panels) from FACS analysis of double positive CD4 and CD8 (DP), single positive CD4 (SP4) and single positive CD8 (SP8) thymocytes. **(b)** Number of red blood cells, reticulocytes, and percentages of lymphocytes, monocytes, and neutrophils was evaluated in blood (between 8-14 weeks). **(c)** Total spleen cell counts and proportions of splenic B, T, CD4^{+}, CD8^{+}, NK, DCs, macrophages and neutrophils cells from FACS analysis (between 8-14 weeks). **(d)** Total thymus cell counts (left panel) and proportions (right panels) from FACS analysis of double positive CD4 and CD8 (DP), single positive CD4 (SP4) and single positive CD8 (SP8) thymocytes. **(e)** Total spleen cell counts and proportions of splenic B, T, NK, DCs, macrophages and neutrophils cells from FACS analysis (between 8-14 weeks). Data are from n=5 (wt/flox), n=9 (flox/flox), n=8 (wt/ko) and n=7 (ko/ko) animals per group (**panel a**), n=6 (control) and n=5 (ko/ko) animals per group (**panel b**), n=6 (wt/flox), n=12 (flox/flox), n=11 (wt/ko) and n=12 (ko/ko) animals per group (**panel c),** n=10 (control) and n=12 (ko/ko) animals per group (**panel d)** and n=15 (control) and n=15 (ko/ko) animals per group (**panel d)**.

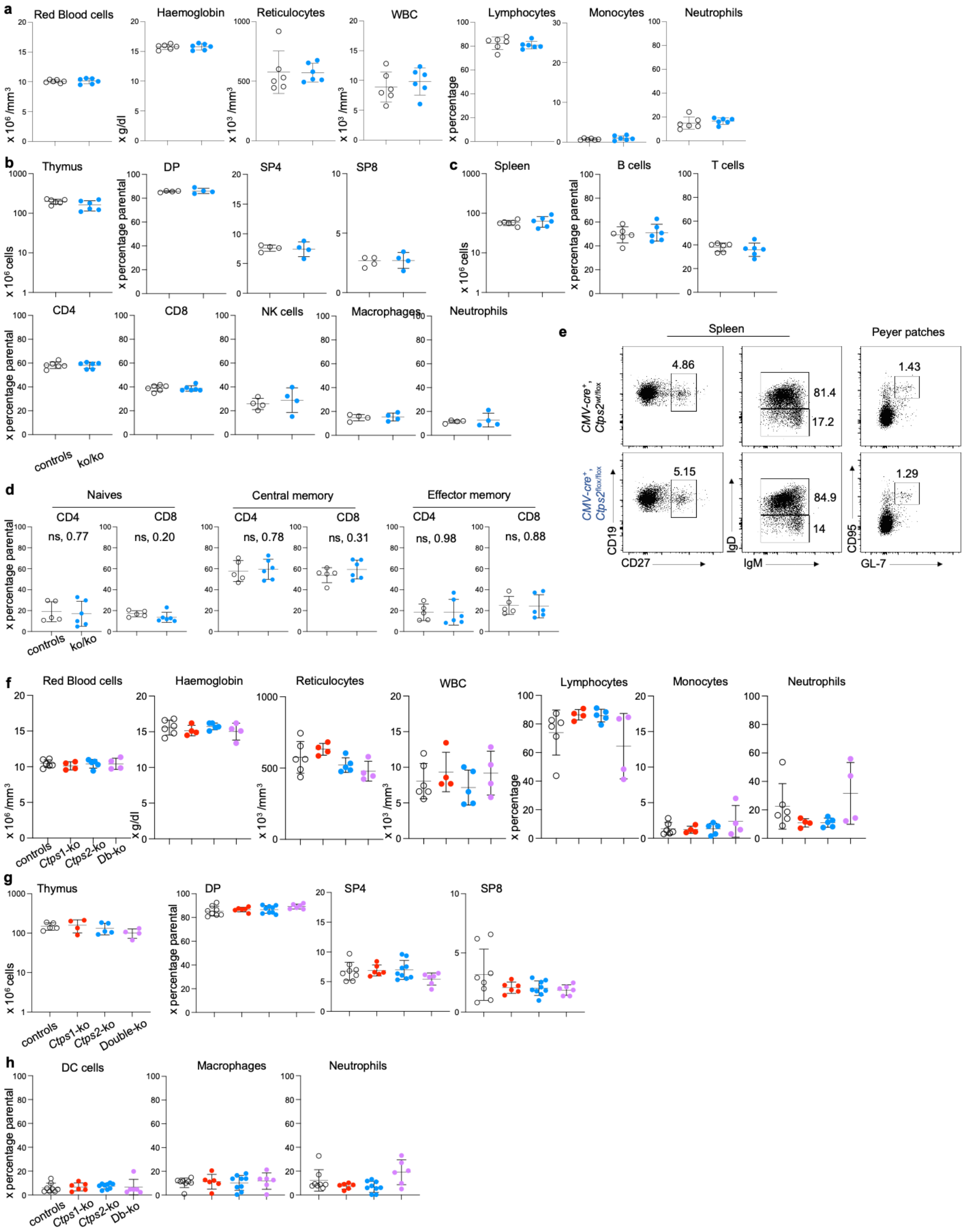

Supplementary Figure 7. **Complementary analyses of *Ctps2* inactivation upon the *CMV* promoter and double *Ctps1* and *Ctps2* inactivation upon the *CD4* promoter.** Complementary analyses of *CMV-Cre* conditional *Ctps2* deficient animals **(a-d)** and *CD4-Cre* conditional *Ctps1* and *Ctps2* deficient animals **(d-g)**.

(a-e) Analyses of haematological and immunological parameters of *CMV-Cre; Ctps2*$^{\text{flox/flox}}$ (*Ctps2*$^{\text{ko/ko}}$) and of control littermate (*Ctps2*$^{\text{wt/flox}}$, *Ctps2*$^{\text{flox/flox}}$ and *Ctps2*$^{\text{wt/ko}}$) animals. **(a)** Red blood cells, haemoglobin levels, reticulocytes, whole blood cells numbers and proportions of lymphocytes, monocytes and neutrophils in blood. **(b)** Total thymus cell counts (left panel) and

proportions (right panels) from FACS analysis of double positive CD4 and CD8 (DP), single positive CD4 (SP4) and single positive CD8 (SP8) thymocytes. **(c)** Total spleen cell counts (left panel) and proportions of B, T, $CD4^+$, $CD8^+$, NK cells, macrophages and neutrophils (right panels). **(d)** Proportions of T cell subsets from FACS analyses after activation. Naive T cells ($CD44^-CD62L^+$), central memory ($CD44^+CD62L^+$), effector memory ($CD44^+CD62L^-$) are shown among both $CD4^+$ and $CD8^+$ T cells. **(e)** Representative dot-plots analysis of spleen memory B cells ($B220^+CD19^+CD27^+$), mature ($B220^+CD19^+IgD^+$), and transitional ($B220^+CD19^+IgD^-$) spleen B cells, left panels. Representative dot-plots analysis of Peyer patches from the intestine, stained with anti-CD95 and anti-GL7, right panels. Representative of four *Ctps2*^{ko} in two independent experiments.

(f-h) Complementary analyses of haematological and immunological parameters of *CD4-Cre*; *Ctps1*$^{wt/flox}$x*Ctps2*$^{wt/flox}$ (*Ctps1*$^{wt/ko}$, *Ctps2*$^{wt/ko}$) empty black circle, *CD4-Cre*; *Ctps1*$^{flox/flox}$x*Ctps2*$^{wt/flox}$ (*Ctps1*$^{ko/ko}$, *Ctps2*$^{wt/ko}$) filled red circle, *CD4-Cre*; *Ctps1*$^{wt/flox}$x*Ctps2*$^{flox/flox}$ (*Ctps1*$^{wt/ko}$, *Ctps2*$^{ko/ko}$) filled blue circle and *CD4-Cre*; *Ctps1*$^{flox/flox}$x*Ctps2*$^{flox/flox}$ (*Ctps1*$^{ko/ko}$, *Ctps2*$^{ko/ko}$) filled purple circle. **(f)** Red blood cells, haemoglobin levels, reticulocytes, whole blood cells numbers and proportions of lymphocytes, monocytes and neutrophils in blood. **(g)** Total thymus cell counts (left panel) and proportions (right panels) from FACS analysis of double positive CD4 and CD8 (DP), single positive CD4 (SP4) and single positive CD8 (SP8) thymocytes. **(h)** Proportions of splenic DC cells, macrophages and neutrophils from FACS analysis. Data are from n=6 (control), n=6 (Ctps2ko) animals per group (**panel a**), n=4 (control), n=4 (Ctps2ko) animals per group (**panel b**), n=6 (control), n=6 (Ctps2ko) animals per group (**panel c**) and n=5 (control), n=6 (Ctps2ko) animals per group (**panel d**). Non-parametric Matt-Whitney two-tailed test was used. Error bars represent the mean ± SD of n=6 (controls), n=4 (Ctps1ko), n=5 (Ctps2ko) and n=4 (DKO) animals per group (**panel f**) and n=8 (controls), n=6 (Ctps1ko), n=11 (Ctps2ko) and n=6 (DKO) animals per group (**panel g,h**).

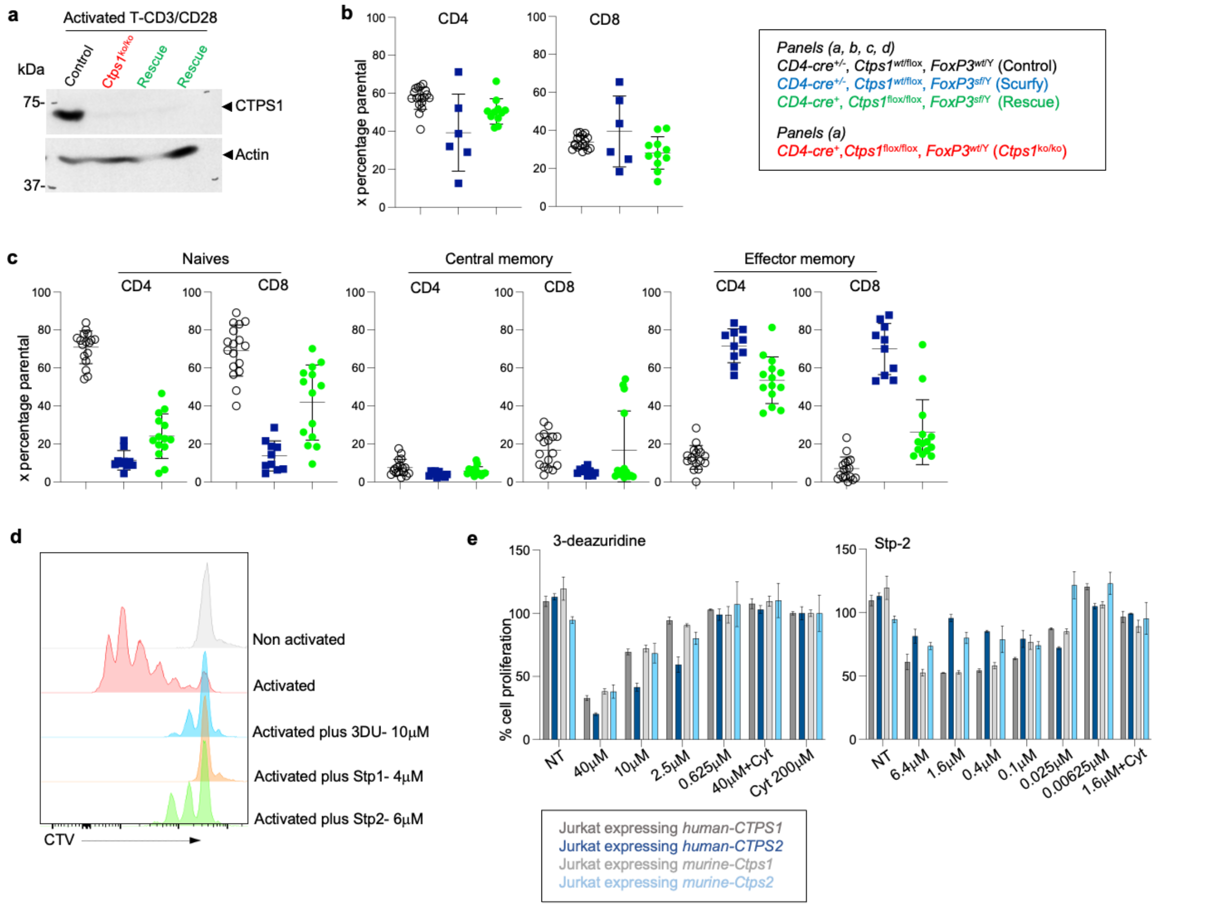

Supplementary Figure 8. **Complementary analyses for the inhibition of *CTPS1* in *Scurfy* mice.**

Complementary analyses *CD4-Cre* conditional *Ctps1* crossed to Scurfy *FoxP3*$^{sf/y}$ animals **(a-c).**

(a) Immunoblots for CTPS1 and Actin expression in lysates of activated T cells. Molecular weights in kDa on the left. **(b)** Graph bars showing proportions of splenic CD4+, CD8+ from FACS analysis. **(c)** Graph bars showing proportions of splenic T cell subpopulations including CD4+, CD8+, naive T cells (CD44^{-}CD62L^{+}), central memory (CD44^{+}CD62L^{+}), effector memory (CD44^{+}CD62L^{-}) from FACS analysis. All shown in both CD4^{+} and CD8^{+} T cells; in *CD4-Cre* conditional *Ctps1* crossed to Scurfy *FoxP3*$^{sf/y}$ animals. **(d)** Analyses of specific Stp-CTPS1 inhibitors on splenic T cells. Enriched spleen T cells were activated by anti-CD3/CD28 plus IL-2 for 3 days and 3-DU (blue) or specific Stp inhibitors (Stp-1 and Stp-2) (orange and green) were added to the culture. Proliferation was assessed by CTV dilution by FACS analysis. **(e)** Dose response of inhibition of proliferation of jurkat cell lines expressing human *CTPS1*, human *CTPS2*, mouse *Ctps1* or mouse *Ctps2*, using 3-DU (left panel) and Stp-2 (right panel) inhibitors. Data are results of triplicates measurements for each condition. Data are from n=18 (control), n=6 (scurfy) and n=11 (rescue) (**panel b**), and of n=17 (control), n=10 (scurfy) and n=14 (rescue) (**panel c**).

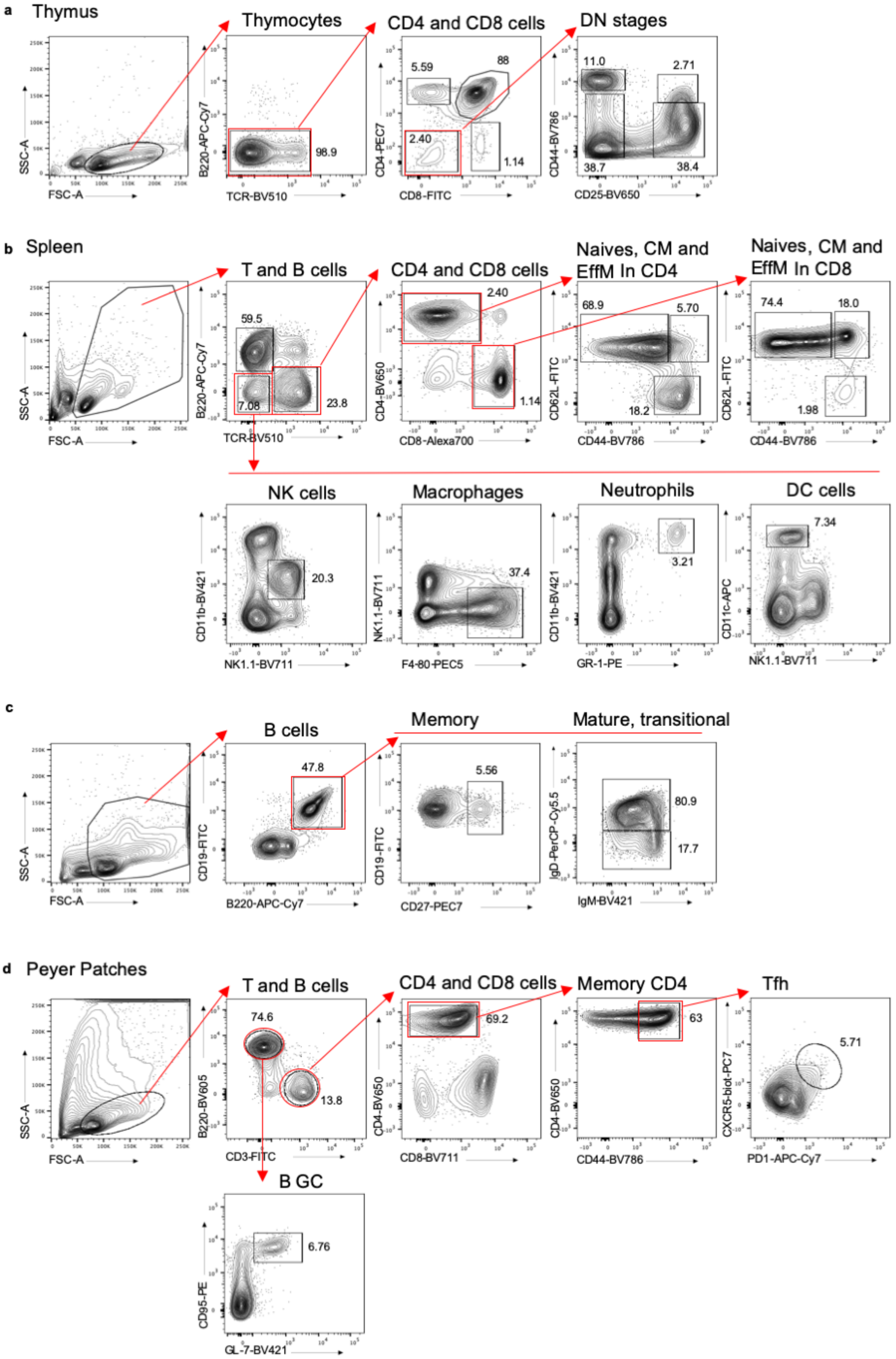
a
Thymus
Thymocytes
CD4 and CD8 cells
DN stages
SSC-A
FSC-A
B220-APC-Cy7
TCR-BV510
98.9
CD4-PEC7
CD8-FITC
5.59
88
2.40
1.14
CD44-BV786
CD25-BV650
11.0
2.71
38.7
38.4
b
Spleen
T and B cells
CD4 and CD8 cells
Naives, CM and EffM In CD4
Naives, CM and EffM In CD8
SSC-A
FSC-A
B220-APC-Cy7
TCR-BV510
59.5
7.08
23.8
CD4-BV650
CD8-Alexa700
2.40
1.14
CD62L-FITC
CD44-BV786
68.9
5.70
18.2
CD62L-FITC
CD44-BV786
74.4
18.0
1.98
NK cells
Macrophages
Neutrophils
DC cells
CD11b-BV421
NK1.1-BV711
20.3
NK1.1-BV711
F4-80-PEC5
37.4
CD11b-BV421
GR-1-PE
3.21
CD11c-APC
NK1.1-BV711
7.34
c
B cells
Memory
Mature, transitional
SSC-A
FSC-A
CD19-FITC
B220-APC-Cy7
47.8
CD19-FITC
CD27-PEC7
5.56
IgD-PerCP-Cy5.5
IgM-BV421
80.9
17.7
d
Peyer Patches
T and B cells
CD4 and CD8 cells
Memory CD4
Tfh
SSC-A
FSC-A
B220-BV605
CD3-FITC
74.6
13.8
CD4-BV650
CD8-BV711
69.2
CD4-BV650
CD44-BV786
63
CXCR5-biot-PC7
PD1-APC-Cy7
5.71
B GC
CD95-PE
GL-7-BV421
6.76

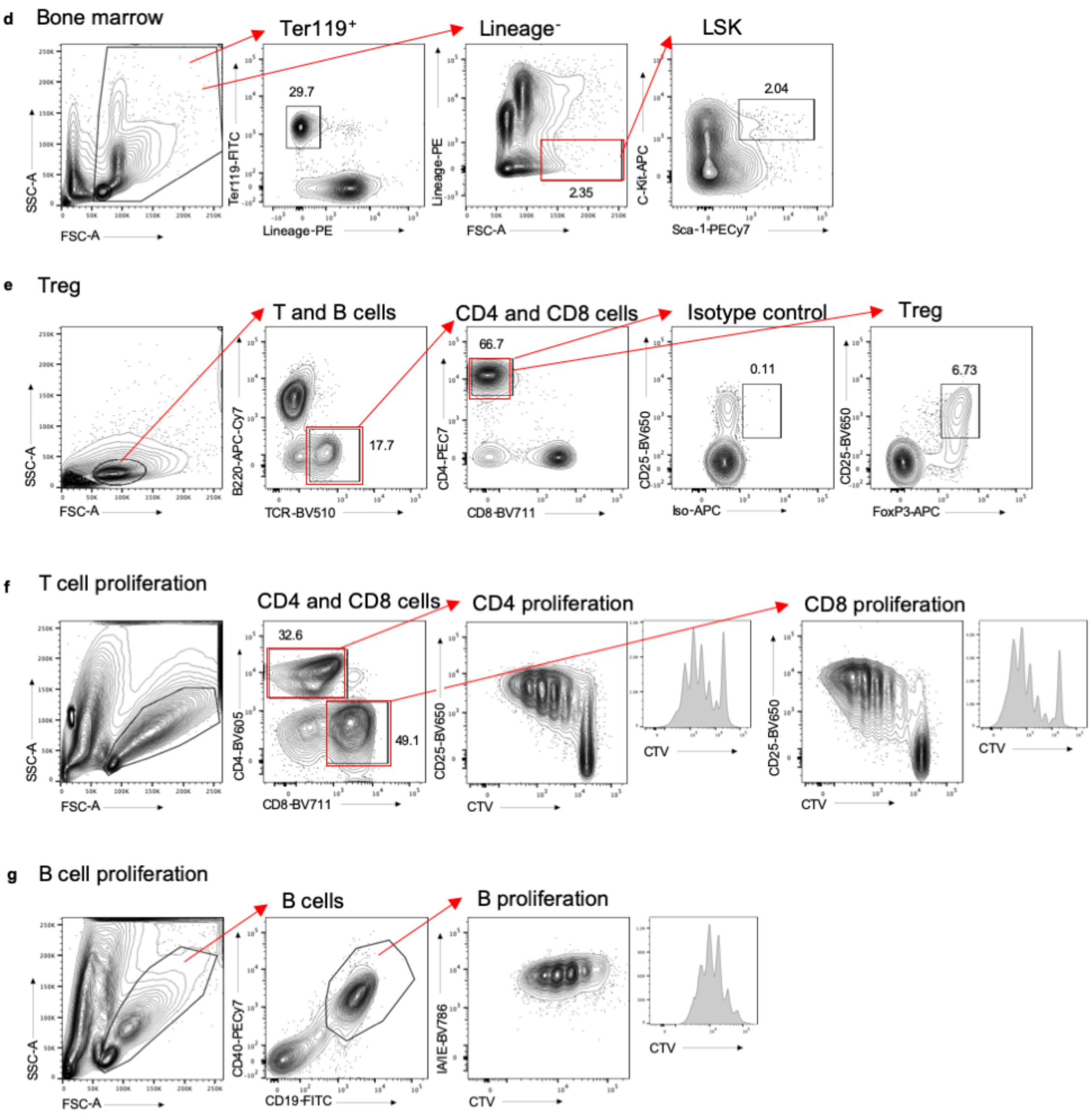

Supplementary Figure 9. **Gating strategy**

(a) Thymus, identification of the different thymic development stages. **(b)** Spleen, identification of the different cell populations and activation status. **(c)** Spleen, specific identification of B cells phenotype. **(d)** Peyers-patches, identification of follicular helper T cells and germinal centre B cells. **(e)** Bone marrow, identification erythrocyte progenitors and haematopoietic stem and multi-potent progenitor (LSK). **(f, g)** Spleen, identification of proliferating CD4 and CD8 T cells **(f)** and B cells **(g)**.

Supplementary Table-1

Guide, matrix and primers used:

CTPS1Ki_F	TAGACATGCCAGAACATAACC	(exon14)
CTPS1Ki_R	TCATAGTTGTAAACTGTCAAAGC	(exon 18)
CTPS1KO_A	CAA ATG GCA CAG GGT TCT GG	
CTPS1KO_D	TCC AGT CCT GGA ATG AAG GG	
CTPS1KO_E	ACT GAT GTA CTT TTC TGC CTG G	
CTPS2KO_A	ATT GGC GCA GGG TGT CTT TTT CC	
CTPS2KO_D	CAT GAG AAA CTC AGT TGA AAC CCA C	
CTPS2KO_E	AGT TGT TAT CCC CAT CCT GGT CTG C	
ViCre_F	ACG GCT GGA TGA GAT AGT GG	
ViCre_R	CAC AGT CAG CAG GTT GGA GA	
CRE_F	GCG GTC TGG CAG TAA AAA CTA TC	
CRE_R	CTC TAC ACC TGC GGT GCT AAC	
R26_F	AAA GTC GCT CTG AGT TGT TAT	
R26_R	GGA GCG GGA GAA ATG GAT ATG	
CRE-ERt2	CCT GAT CCT GGC AAT TTC G	
SCURFY_F	AGG AAG ATA AGC GAT GAG GG	
SCURFY_R	GAA GGA ACT ATT GCC ATG GC	

For CRISPR-Cas9:
Guide oligo: TAACCCTTCTCAATTCCACAGGG
Matrix:
CGTGCTGCATATAACCGTGGTTGCAAAGGTGGGGAAGTAAAGTGAGTCCCTGACGGGAGCTG
AGTAACtCTTCTgAATTCtACA**C**GGACACTTACAGTGACAGAAGCGGGAGCAGCTCCCCCGA
CTCGGAAATCACTGAACTCAAGTTTCCATCAATAAG

Supplementary Table-2

List of antibodies used in the study:

**Antigen**	**Clone number**	**Dilution**
anti-TCR	clone H57-597	1/200
anti-CD3	clone 145-2C11	1/200
anti-CD4	clone RMA4-5	1/200
anti-CD8	clone 53-6.7	1/200
anti-CD11b	clone Ml/70	1/200
anti-CD11c	clone N418	1/200
anti-CD19	clone 6D5	1/200
anti-B220	clone RA3-6B2	1/150
anti-CD25	clone PC61	1/200
anti-CD27	clone LG3A10	1/200
anti-CD44	clone IM7	1/200
anti-CD62L	clone MEL-14	1/200
anti-CD40	clone 3/23	1/200
anti-lA/IE	clone M5/114.15.2	1/200
anti-GR1	clone RB6-8C5	1/200
anti-F4/80	clone BM8	1/200
anti-Ter119	clone Ter119	1/200
anti-Scal	clone D7	1/200
antiCD117	clone 2B8	1/200
anti-NKl.1	clone PK136	1/200
anti-CD95	clone DX2	1/100
anti-GL-7	clone GL-7	1/150
antiCXCR5	clone Ll38D7	1/200
anti-PD-1	clone 29F.1A12	1/200
anti-lgM	clone RMM-1	1/200
anti-lgD	clone 11-26c2a	1/200
anti-isotype IgG2a	clone eBR2a	1/200
anti-FoxP3	clone FJK-16S	1/200

All the antibodies for cytofluorimetry were purchased from BD-Bioscience, BioLegend, e-Bioscience.
The antibodies were conjugated to: Fluorescein isothiocyanate (FITC), Peridinin-chlorophyll-cyanin5.5 (PerCP-Cy5.5), R-phycoerythrin (PE), Phycoerythrin-cyanin5 (PE-Cy5), Phycoerythrin-cyanin7 (PE-Cy7), Allophycocyanin (APC), Allophycocyanin-Cyanin7 (APC-Cy7), Alexa-700, Brilliant Violet 421 (BV421), Brilliant Violet 510 (BV510), Brilliant Violet 605 (BV605), Brilliant Violet 650 (BV650), Brilliant Violet 711 (BV711), Brilliant Violet 785 (BV785).
For immunoblotting, the following primary antibodies were used at a 1/1000 dilution: rabbit monoclonal anti-CTPSl EPR8086 (Abcam ab133743) or rabbit polyclonal anti-CTPS2 (C-ter) (Abcam ab190462) and at a 1/5000 dilution: mouse monoclonal anti-actin (Santa-Cruz-4778). Secondary antibodies from Cell Signalling were used at a 1/10000 dilution: anti-rabbit HRP-linked Ab (7074S) and anti-mouse HRP-linked Ab (7076S).
