# Supplementary material for: Inactivation of cytidine triphosphate synthase 1 prevents fatal auto-immunity in mice
Source: Nat Commun. 2024 Mar 4;15:1982. doi: 10.1038/s41467-024-45805-y (PMC10912214; doi:10.1038/s41467-024-45805-y)
Supplement: Supplementary file 3 — Reporting Summary [file 41467_2024_45805_MOESM3_ESM.pdf]

nature portfolio

Corresponding author(s): Claire Soudais and Sylvain Latour

Last updated by author(s): Dec 14, 2023

Reporting Summary

Nature Portfolio wishes to improve the reproducibility of the work that we publish. This form provides structure for consistency and transparency in reporting. For further information on Nature Portfolio policies, see our Editorial Policies and the Editorial Policy Checklist.

Statistics

For all statistical analyses, confirm that the following items are present in the figure legend, table legend, main text, or Methods section.

n/a	Confirmed	
☐	☒	The exact sample size (*n*) for each experimental group/condition, given as a discrete number and unit of measurement
☐	☒	A statement on whether measurements were taken from distinct samples or whether the same sample was measured repeatedly
☐	☒	The statistical test(s) used AND whether they are one- or two-sided<br>*Only common tests should be described solely by name; describe more complex techniques in the Methods section.*
☒	☐	A description of all covariates tested
☒	☐	A description of any assumptions or corrections, such as tests of normality and adjustment for multiple comparisons
☐	☒	A full description of the statistical parameters including central tendency (e.g. means) or other basic estimates (e.g. regression coefficient) AND variation (e.g. standard deviation) or associated estimates of uncertainty (e.g. confidence intervals)
☐	☒	For null hypothesis testing, the test statistic (e.g. *F*, *t*, *r*) with confidence intervals, effect sizes, degrees of freedom and *P* value noted<br>*Give P values as exact values whenever suitable.*
☒	☐	For Bayesian analysis, information on the choice of priors and Markov chain Monte Carlo settings
☒	☐	For hierarchical and complex designs, identification of the appropriate level for tests and full reporting of outcomes
☒	☐	Estimates of effect sizes (e.g. Cohen's *d*, Pearson's *r*), indicating how they were calculated

Our web collection on statistics for biologists contains articles on many of the points above.

Software and code

Policy information about availability of computer code

Data collection	The following sofwares were used for data collection,<br>-Flow cytometry : BD FACSDlva software version 9.0- Becton Dickinson & Compagny (BD) on<br>-LSR Fortessa X20 and ARIA II (Becton Dickinson)<br>-Sanger sequencing : 3500 Series Data collection software version 2 - Thermofischer Scientific
Data analysis	The following software were used for data analysis,<br>- FlowJo 2 (Becton Dickinson) (10.7.1)<br>- GraphPad Prism (9.4.1)<br>- ImageJ (Fiji) (2.9.0)<br>- Imaris software (9.9)<br>- 4peaks (1.8)<br>- Oligonucleotides for CRISPR-Cas9 were designed with http://crispor.tefor.net/.

For manuscripts utilizing custom algorithms or software that are central to the research but not yet described in published literature, software must be made available to editors and reviewers. We strongly encourage code deposition in a community repository (e.g. GitHub). See the Nature Portfolio guidelines for submitting code & software for further information.

Data

Policy information about availability of data

All manuscripts must include a data availability statement. This statement should provide the following information, where applicable:

- Accession codes, unique identifiers, or web links for publicly available datasets
- A description of any restrictions on data availability
- For clinical datasets or third party data, please ensure that the statement adheres to our policy

All materials used in this study are available to researchers following appropriate standard transfer agreement. Requests and correspondence should be addressed to Claire Soudais or Sylvain Latour.

Research involving human participants, their data, or biological material

Policy information about studies with human participants or human data. See also policy information about sex, gender (identity/presentation), and sexual orientation and race, ethnicity and racism.

Reporting on sex and gender	N/A
Reporting on race, ethnicity, or other socially relevant groupings	N/A
Population characteristics	N/A
Recruitment	N/A
Ethics oversight	N/A

Note that full information on the approval of the study protocol must also be provided in the manuscript.

Field-specific reporting

Please select the one below that is the best fit for your research. If you are not sure, read the appropriate sections before making your selection.

☒ Life sciences ☐ Behavioural & social sciences ☐ Ecological, evolutionary & environmental sciences

For a reference copy of the document with all sections, see nature.com/documents/nr-reporting-summary-flat.pdf

Life sciences study design

All studies must disclose on these points even when the disclosure is negative.

Sample size	No statistical method was used to predetermine sample size. Sample size was chosen as a result of previous experience regarding variability in mouse models, experimental set-up and standards in the field, according to literature monitoring. Experiments were repeated to assure statistical differences.
Data exclusions	Some histological samples were excluded in case of identified freezing problem.
Replication	All experiments were carried out at least in three independant biological replicates.<br>Each point on all the figures and supplemenrtary represent values from individual animals.
Randomization	For all experiments, animais were genotyped and litter mate with all the genotypes being used in the experiments. Heterozygous breedings were maintained for that purpose.
Blinding	The investigators were blinded to group allocation during data collection and/or analysis. All data were collected according to animal number and not to genotype, further processing of the data was related to the animal number.

Reporting for specific materials, systems and methods

We require information from authors about some types of materials, experimental systems and methods used in many studies. Here, indicate whether each material, system or method listed is relevant to your study. If you are not sure if a list item applies to your research, read the appropriate section before selecting a response.

Materials & experimental systems

n/a	Involved in the study	
☐	☒	Antibodies
☐	☒	Eukaryotic cell lines
☒	☐	Palaeontology and archaeology
☐	☒	Animals and other organisms
☒	☐	Clinical data
☒	☐	Dual use research of concern
☒	☐	Plants

Methods

n/a	Involved in the study	
☒	☐	ChIP-seq
☐	☒	Flow cytometry
☒	☐	MRI-based neuroimaging

Antibodies

Antibodies used

The following mouse antibodies were conjugated to fluorescein isothiocyanate (FITC), R-phycoerythrin (PE), phycoerythrin-cyanin5 (PE-Cy5), phycoerythrin-cyanin7 (PE-Cy7), Peridinin-chlorophyll-cyanin5.5 (PerCP-Cy5.5), allophycocyanin (APC), allophycocyanin-Cyanin7 (APC-Cy7), alexa-700, Brilliant Violet 421 (BV421), Brilliant Violet 510 (BV510), Brilliant Violet 605 (BV605), Brilliant Violet 650 (BV650), Brilliant Violet 711 (BV711), Brilliant Violet 785 (BV785): anti-TCR (clone H57-597), anti-CD4 (clone RMA4-5), anti-CD8 (clone 53-6.7), anti-CDllb (clone Ml/70), anti-CDllc (clone N418), anti-CD19 (clone 6D5), anti-B220 (clone RA3-6B2), anti-24 (clone Ml/69), anti-25 (clone PC61), anti-27 (clone LG3A10), anti-CD44 (clone IM7), anti-62L (clone MEL-14), anti-CD95 (clone DX2), anti-CD117 (clone 2B8), anti-GRl (clone RB6-8C5), anti-F4/80 (clone BM8), anti-Ter119 (clone Ter119), anti-Scal (clone D7), anti-NKP46 (clone 29Al.4), anti-NKl.1 (clone PK136), anti-Ter119 (clone Ter119), anti-lA/IE (clone M5/114.15.2), anti-GL-7 (clone GL-7), anti-CXCR5 (clone Ll38D7), anti-PD-1 (clone 29F.1A12), anti-Ter119 (clone Ter119), anti-lgM (clone RMM-1), anti-lgD (clone 11-26c2a). For intracellular FoxP3 staining, samples were blocked with Trusta in FcXtm PLUS, surfaced labelled then, isotype control-APC (Rat lgG2a, RTK2758) or anti-FoxP3-APC (FJK-16S, eBioScience) antibodies were used with the staining buffer set and protocol from eBioscience. For immunoblotting, the following primary antibodies were used: rabbit monoclonal anti-CTPSl EPR8086 (Abcam ab133743) or rabbit polyclonal anti-CTPS2 (C-ter) (Abcam ab190462) and mouse monoclonal anti-actin (Santa-Cruz-4778).Secondary antibodies from Cell Signalling were used at a 1/10000 dilution: anti-rabbit HRP-linked Ab (7074S) and anti-mouse HRP-linked Ab (7076S).

Validation

All these antibodies were purchased from BD, BioLegend, e-Bioscience, Abcam, Santa-Cruz and Cell Signalling. They were validated by the manufacturer via FACS or immunoblotting. Thes data are available on manufacturer's websites (https://www.bdbiosciences.com/en-fr; https://www.biolegend.com/fr-fr; https://www.thermofisher.com/fr/fr/home/life-science/antibodies.html, https://www.abcam.com/nav/primary-antibodies, https://www.scbt.com/browse/antibodies/_/N-med3ky, https://www.cellsignal.com/). Additional validation is provided for antibodies used in iwestern blotting experiments by data shown the figures and extended figures in the manuscript.

Eukaryotic cell lines

Policy information about cell lines and Sex and Gender in Research

Cell line source(s)

Jurkat cell were purchase from ATCC (RRID:CVCL_0065). Jurkat cell line was established from the peripheral blood of a 14-year-old, male, acute T-cell leukemia patient.

Authentication

Knock-out CTPS1 and CTRPS2 Jurkat cells line were sequenced and protein presence was evaluated according to https://doi.org/10.26508/lsa.202302066.

Mycoplasma contamination

Jurkat cells were tested and negative for mycoplasma before used.

Commonly misidentified lines
(See ICLAC register)

None

Animals and other research organisms

Policy information about studies involving animals; ARRIVE guidelines recommended for reporting animal research, and Sex and Gender in Research

Laboratory animals

All animais were analysed between 6 to 12 weeks of age. Ctpsl and Ctps2 ES cells, purchase from KOMP (ID:89620) and EUCOMM (ID:114201) respectively, were injected into blastocysts to generate founders. Chimeric animais were obtained and bred for germ line transmission. Founders were further backcrossed to C57B1/6J wild type mice. These animais were further bred to transgenic mice expressing the flipase transgene to delete the KOMP cassette and generate animals with flox alleles. Homozygous flox animais were then crossed to specific CRE transgenic animals. The following CRE transgenic mouse were used: Tg Flipase JAX stock #012930, CMV-Cre JAX stock #006054, Vav-icre JAX stock #008610, CD8-Cre JAX stock #008766, CD4-Cre JAX stock #017336, Cre-ERT2 JAX stock #008463. The Scurfy mice JAK stock#004088 line was maintained by backcrossing on a B6.RAGl KO background #002216.
To obtain genetically modified mice carrier the same mutation as human, mouse mutated allele was generated through CRISPR/Cas9 mediated HR in C57BL/6J mouse zygotes using CRISPR ribonucleoprotein (RNP) complex direct delivery in pronuclei by electroporation.
All animals are housed in cages of a maximum of 5 animals. Animal were housed in individual ventilated cages with enrichment (with cotton wool wooden sticks), under specific and opportunistic pathogen-free (SOPF) conditions. Animals were fed standard chow diet

	ad libitum, and kept under ambient temperature (21–22 °C) and 50–60% humidity, with 12 h–12 h on-off light cycle. All experiments are done accoprding to the principles of the 3Rs (Replacement, Reduction and Refinement).
Wild animals	No wild type animals were used in this study.
Reporting on sex	Both male and female mice were used in this study.
Field-collected samples	No field collected samples were used in this study.
Ethics oversight	All mouse experiments were performed in accordance with European Union (EU) Directive 2010/63/EU. Animal procedures were approved by the animal committee of the University of Paris Descartes (Paris, France) and the Ministry of Higher Education, Research and Innovation (APAFIS#32465).

Note that full information on the approval of the study protocol must also be provided in the manuscript.

Plants

Seed stocks	N/A
Novel plant genotypes	N/A
Authentication	N/A

Flow Cytometry

Plots

Confirm that:

☐ The axis labels state the marker and fluorochrome used (e.g. CD4-FITC).

☐ The axis scales are clearly visible. Include numbers along axes only for bottom left plot of group (a 'group' is an analysis of identical markers).

☒ All plots are contour plots with outliers or pseudocolor plots.

☒ A numerical value for number of cells or percentage (with statistics) is provided.

Methodology

Sample preparation	Cell suspensions were prepared from thymii and spleen by mechanical disruption on cell strainers 70 microm and resuspended on ice in FACS buffer (PBS containing 2% FCS). Prior to staining, all samples were blocked with TruStain FcXtm PLUS (anti-mouse CD16/32, clone S17011E) from BioLegend in FACS buffer for 15 min on ice.
Instrument	All data were collected on LSR-Fortessa X20 cytometer (from BD Biosciences)<br>BD FACSAria-11 SORP sorter - Becton Dickinson & Compagny (BD)
Software	For acquisition: BD FACSDlva software version 9.0- Becton Dickinson & Compagny (BD)<br>For analysis : Flowcytometry data were processed using FlowJO software version 10.8.0 (Treestar).
Cell population abundance	Enough cells in the final gate were acquired to ensure statistical differences.
Gating strategy	The single cells were plotted against side scatter parameter (SSC) versus forward scatter (FSC) parameter. The events with very low SSC and FSC gating were removed, they correspond to debris, cell fragments or pyknotic cells. Gating strategy for all cell populations described in the paper is precisely shown in supplementary data Figure 9.

☒ Tick this box to confirm that a figure exemplifying the gating strategy is provided in the Supplementary Information.
